# Bioinformatics Identification of Drug Resistance-Associated Gene Pairs in *Mycobacterium tuberculosis*

**DOI:** 10.3390/ijms17091417

**Published:** 2016-08-27

**Authors:** Ze-Jia Cui, Qing-Yong Yang, Hong-Yu Zhang, Qiang Zhu, Qing-Ye Zhang

**Affiliations:** Hubei Key Laboratory of Agricultural Bioinformatics, College of Informatics, Huazhong Agricultural University, Wuhan 430070, China; zejia_cui@outlook.com (Z.-J.C.); yqy@mail.hzau.edu.cn (Q.-Y.Y.); zhy630@mail.hzau.edu.cn (H.-Y.Z.)

**Keywords:** *Mycobacterium tuberculosis*, drug resistance, gene pair, GBOOST

## Abstract

Tuberculosis is a chronic infectious disease caused by *Mycobacterium tuberculosis* (*Mtb*). Due to the extensive use of anti-tuberculosis drugs and the development of mutations, the emergence and spread of multidrug-resistant tuberculosis is recognized as one of the most dangerous threats to global tuberculosis control. Some single mutations have been identified to be significantly linked with drug resistance. However, the prior research did not take gene-gene interactions into account, and the emergence of transmissible drug resistance is connected with multiple genetic mutations. In this study we use the bioinformatics software GBOOST (The Hong Kong University, Clear Water Bay, Kowloon, Hong Kong, China) to calculate the interactions of Single Nucleotide Polymorphism (SNP) pairs and identify gene pairs associated with drug resistance. A large part of the non-synonymous mutations in the drug target genes that were included in the screened gene pairs were confirmed by previous reports, which lent sound solid credits to the effectiveness of our method. Notably, most of the identified gene pairs containing drug targets also comprise Pro-Pro-Glu (PPE) family proteins, suggesting that PPE family proteins play important roles in the drug resistance of *Mtb*. Therefore, this study provides deeper insights into the mechanisms underlying anti-tuberculosis drug resistance, and the present method is useful for exploring the drug resistance mechanisms for other microorganisms.

## 1. Introduction

Tuberculosis (TB) is one of most dangerous chronic infectious diseases and it is caused by *Mycobacterium tuberculosis* (*Mtb*) infection. *Mtb* is typically transmitted by aerosols and reaches the lungs, where macrophages and other immune cells are recruited during the early innate response to infection [1,2]. TB has become a significant threat to public health. The World Health Organization (WHO) Global Tuberculosis Report indicated that in 2014, approximate 9.6 million people were carriers of *Mtb*, and 1.5 million people died of TB [3]. In the early 20th century, with the development of effective antibiotics and vaccines, the deaths caused by *Mtb* were controlled. However, TB became an ongoing threat to global health in the late 20th century, and the current epidemic is fueled by Human Immunodeficiency Virus (HIV) coinfection and an increasing incidence of drug-resistant strains of *Mtb* [4]. The extensive treatment courses result in poor compliance and drug resistance, and the emergence of multidrug-resistant strains has become a serious public health threat and represents a new challenge in TB control.

The first TB therapy, streptomycin (STR), was discovered and used to combat TB in 1944 [5]. Soon afterwards, many more anti-TB agents were developed. Drugs employed in TB treatment are classified into five groups, as shown in Table 1 [6]. Group 1 represents the first-line oral agents, which include isoniazid (INH), rifampicin (RMP), ethambutol (EMB), pyrazinamide and rifabutin. Group 2 includes the injectable agents, which comprise aminoglycoside antibiotics (kanamycin (KAN); amikacin; STR) and peptide drugs (capreomycin (CPM)). Group 2 drugs can be used together with group 3 fluoroquinolone drugs, which include ofloxacin, levofloxacin, moxifloxacin and gatifloxacin. Group 4 drugs include the oral bacteriostatic second-line agents: *p*-aminosalicylic acid, cycloserine, terizidone, ethionamide (ETH) and prothionamide. Group 5 comprises agents with unclear efficacy and primarily includes clofazimine, linezolid and amoxicillin. The group 5 drugs can be used only when none of other groups of drugs can be used for TB treatment [7].

In general, the mechanism of anti-TB drugs can be divided into four types: (i) inhibition of RNA synthesis; (ii) inhibition of protein synthesis; (iii) inhibition of cell wall biosynthesis; and (iv) interference with the synthesis of cell membranes [8]. INH is a widely used first-line drug to treat TB and is a prodrug susceptible to oxidative reactions catalyzed by catalase-peroxidase (KatG). The formed activated isonicotinoyl-NAD adduct binds to InhA (enoyl-acyl carrier protein reductase) and inhibits the biosynthesis of mycolic acids, which are components of the mycobacterial cell wall, to induce cell lysis [9]. RMP is another first-line drug for the treatment of TB. RMP interferes with DNA-dependent RNA synthesis by inhibiting bacterial DNA-dependent RNA polymerase [8]. EMB is one of the first-line drugs used in treating TB, and the mechanism of EMB is the inhibition of the transfer of arabinogalactan [10]. The second-line anti-TB injectable drugs, which include STR, KAN and CPM, are aminoglycosides. These drugs exhibit similar mechanisms of antibacterial and metabolic activity by blocking the initiation of protein synthesis [11]. Ofloxacin (OFX) belongs to the fluoroquinolone class of drugs that inhibit DNA gyrase (topoisomerase II) and topoisomerase IV, resulting in microbial death [10]. ETH is an oral bacteriostatic second-line agent and is a structural analog of INH. The two drugs exhibit a similar mechanism of action by inhibiting mycolic acid biosynthesis [12].

Due to the extensive use and long-term treatment with the first-line and second-line anti-TB drugs, strains of *Mtb* resistant to anti-TB agents have emerged and increased rapidly. The general anti-TB drug resistance-associated genetic regions are summarized and presented in Table 2 [13]. Drug resistance patterns may vary widely from single-drug to multiple-drug resistance, and most transmissible drug resistance is associated with multiple genetic mutations. Therefore, it is critical to seek out and clarify the resistance mechanism of anti-TB agents and to develop a rapid and comprehensive approach to tackle the current ominous situation [14].

Recently, many research groups have conducted whole genome sequencing on *Mtb* strains with a range of resistance profiles and have identified some new mutations in genes that are significantly linked with *Mtb* drug resistance [15,16]. However, the conventional method does not take gene-gene interactions into account. Accumulating evidence reveals that the emergence of transmissible drug-resistant *Mtb* is the result of a multitude of additional genetic mutations, many of which interact [17]. Therefore, in this paper we performed a bioinformatics analysis to identify gene pairs associated with *Mtb* drug resistance.

## 2. Results and Discussion

All of the interactions of Single Nucleotide Polymorphism (SNP) pairs obtained by GBOOST and the screened corresponding gene pairs from the two datasets are predicted (Tables S1 and S2). Because the mutations of target genes are of most significance in *Mtb* drug resistance [18], we primarily focused on the gene pairs that contained at least one target gene. The drug target information is from the DrugBank database and is listed in Table S3. In dataset 1, *katG*-*PPE54*, *rpoB*-*PPE54* gene pairs associated with INH resistance and RMP resistance were identified, respectively (Table 3). In dataset 2, *embA-PPE68*, *embB-PPE54* gene pairs associated with EMB resistance were detected, and *katG*-*PPE54*, *katG-transcriptional regulator*, *katG-ccsA*, *katG-fdxB* and *katG-oxidoreductase* gene pairs associated with ETH-resistance were discovered (Table 4).

**Figure 1:**
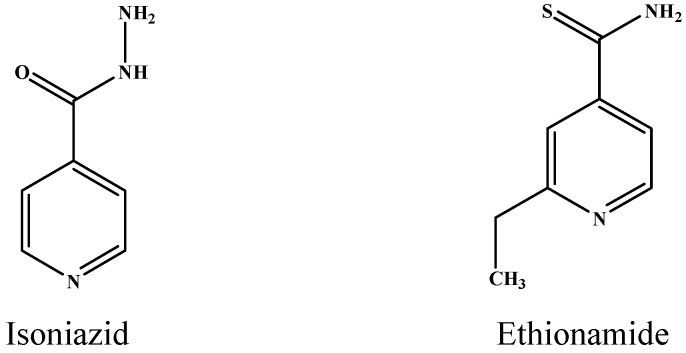
The structures of isoniazid and ethionamide.

To validate the effectiveness of the screening procedure, we sorted out all of the non-synonymous mutations in the drug targets. Overall, five non-synonymous mutations were found in drug targets; three mutations have experimental or theoretical evidence, and the other two are new discoveries (Table 5). One non-synonymous mutation R463L in the *katG* target gene associated with INH resistance was screened out in dataset 1 (Table 5). The R463L SNP site of *katG* was predicted to generate resistance for INH, which had been verified by previous experiments [19]. ETH resistance-associated mutations Y155C and R463L of the *katG* target gene were sorted from gene pairs of dataset 2, as shown in Table 5. The Y155C mutation was consistent with the results of Zhang et al. [16]. The R463L mutation was consistent with the results of INH resistance-associated mutations in dataset 1. ETH is a structural analog of INH (Figure 1). The structural similarity and the existence of cross-resistant phenotypes have suggested that these two drugs share common molecular targets, i.e., *inhA* and *katG* [20]. Therefore, we believe that the R463L mutation would lead to the *Mtb* resistance to ETH. Two EMB resistance-associated mutations, N399T and G406S, of the *embB* target gene were selected. The G406S mutation was consistent with the results of Zhang et al. and was verified by previous experiments [21]. Comparison with the results of Zhang et al. indicated that the N399T mutation was a new site identified by our method, possibly because the previous study did not consider the joint mutation of gene pairs, and the new mutation site is worth experimental verification.

**Figure 2:**
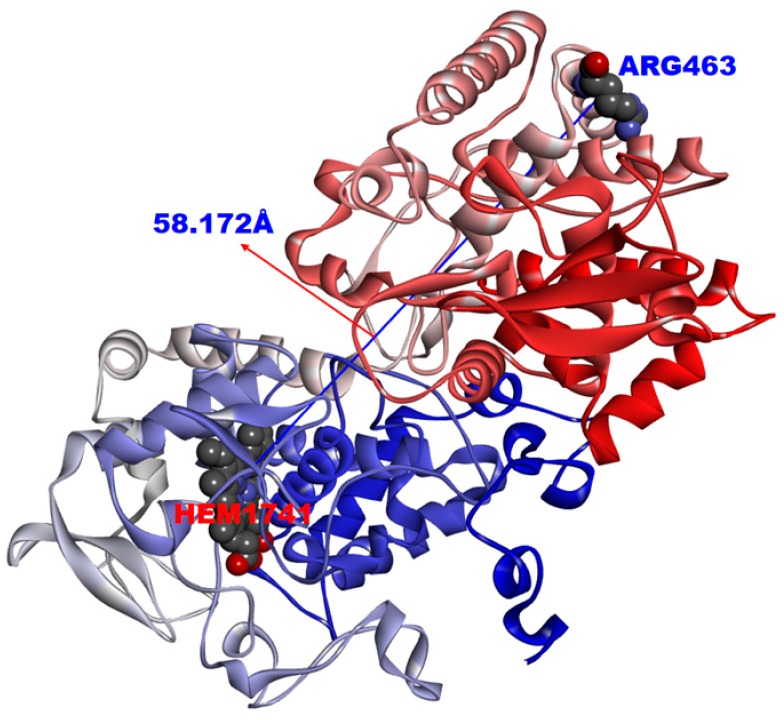
The distance of the active site of the KatG target and Arg463 residue (the distance was approximately 58.172 Å, far from drug binding site).

Interestingly, according to the research report of Phelan et al., there is a strong association between the (shorter) distance of the mutation to the ligand in the protein structure and INH resistance (greater minimum inhibitory concentrations (MIC) values), and the distance between the mutation location and drug binding site was not greater than 9.93 Å [15]. The R463L mutation was not reported in their results. The crystal structure of the KatG protein has been reported (PDB code: 2CCA) [22], and structural analysis revealed that the mutation of R463L is located far from the drug binding site (the distance was approximately 58.172 Å, much greater than 9.93 Å), as shown in Figure 2. This result also lends credit to the present method, which can identify the resistance mutations that far from the drug binding site.

As shown in Table 3 and Table 4, more than 60% of the gene pairs contain one drug target and one PPE family protein (i.e., PPE68 and PPE54). PPE family proteins are unique to mycobacteria and are particularly abundant in pathogenic mycobacteria but are absent from the vaccine strains of Bacille Calmette-Guérin (BCG) [23]. Members of the PPE families are characterized by the presence of a conserved Pro-Pro-Glu (PPE) motif in their N-terminal region [24]. The literatures reported that most of the PPE proteins are located on the cell surface, and PPE proteins have been found to play important roles in generating antigenic variation and immune evasion [25,26,27]. The mutation of PPE protein results in failure to activate the body’s immune response [23]. PPE68 is a member of the PPE protein family and is a membrane protein expressed on the surface of *Mtb* [28,29]. PPE68 is primarily present in the cell wall of *Mtb* and is an immunogenic protein. Experiments confirmed that PPE68 cell surface proteins can induce a specific cellular immune response in mice [30]. Therefore, we infer that the changes in the protein structure caused by mutations would lead to the loss of function of PPE68. However, the biological functions for PPE54 remain elusive. Given that drug effects are associated with immunity and the fact that most of the gene pairs included one drug target and PPE family proteins, the present results suggest that PPE proteins play an important role in *Mtb* drug resistance, which is of apparent interest for future explorations.

In addition, *katG-transcriptional regulator*, *katG*-*ccsA*, *katG*-*fdxB* and *katG*-*oxidoreductase* gene pairs associated with drug resistance were also predicted. Limited by the existing information, the hidden mechanisms of these genes underlying drug resistance are unclear.

## 3. Materials and Methods

### 3.1. Data Source and SNP Calling

In this study, two sets of data were analyzed. The first set of data contained 127 samples of *Mtb*, which included 34 EMB-, 65 INH-, 53 RMP- and 45 STR-resistant strains. A total of 38 pan-susceptible strains of *Mtb* were involved in this dataset. All sequencing data of the first set were downloaded from TDR TB Strain Bank (ENA accession number: PRJEB11653) [15]. All the data of the first set were divided into four groups according to the different resistant strains for the following data analysis, as did by the previous study [15]. The second set of data contained 161 samples of *Mtb*, and all sequencing data were downloaded from the NCBI Sequence Read Archive (SRA) (SRA065095), which included 22 CPM-, 69 EMB-, 36 ETH-, 117 INH-, 21 KAN-, 54 OFX-, 117 RMP- and 83 STR-resistant strains [16]. And a total of 44 pan-susceptible strains of *Mtb* were involved in this set. Similarly, all the data of the second set were divided into eight groups according to the type of resistant strains for the following data analysis, as did by the prior research [16].

All of the downloaded sequencing data were treated with Trimmomatic v0.32 software [31] to remove or truncate reads of low quality (parameter: LEADING: 3; TRAILING: 3; MINLEN: 36; SLIDINGWINDOW: 4:20). High quality reads were then mapped to the *Mtb* H37Rv reference genome (GenBank accession: AL123456.3) using the Bowtie2 v2.2.6 [32] with default parameters. Sequencing filtering and SNP calling were performed by SAMtools v1.2 [33] and VarScan v2.4 [34] (parameters: *min-coverage* = 10; *min-freq-for-hom* = 0.95; *min-avg-qual* = 20), and SNPs with a minor allele frequency (MAF) <0.01 [35] were presumed to be sequencing errors and were discarded. Finally, we used PLINK v1.07 software [36] to filter phylogenetically related SNPs that are likely to influence the gene pair identification (parameter: *indep-pairwise* = 10, 5, 0.5) [37]. This procedure can exclude the gene pairs that could simply result from the strains genotype.

### 3.2. SNP Pair and Gene Pair Calculations

Gene-gene interactions have long been recognized to be fundamentally important in understanding the genetic causes of complex disease trait. GBOOST is a software package identifying gene-gene interactions in genome-wide case-control studies [38]. In this study, we used GBOOST software (parameters: *wm* = GPU; *st* = 12.838; *tt* = 12.838) to detect SNP-SNP interactions on risk of drug resistance. GBOOST adopts a *chi-square* test method to examine the interaction effect between two SNPs and phenotypes. We set the threshold to *p* < 0.005 (χ^2^ > 12.838).

**Figure 3:**
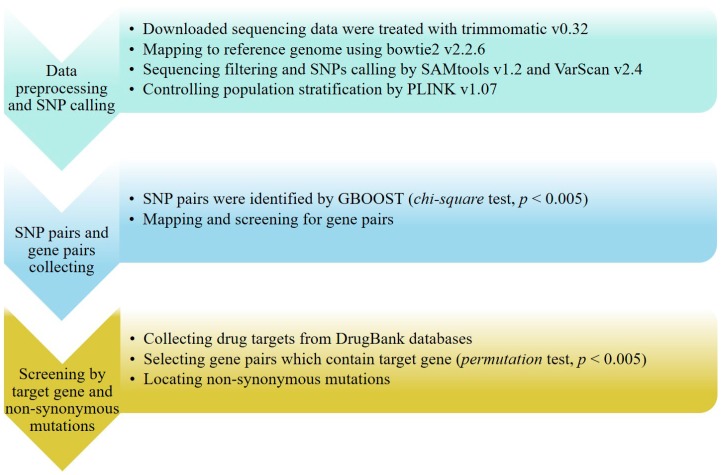
The workflow of the method used in this study.

Taking into account that the mutation in a target gene is a key factor for drug resistance, the SNP pairs obtained by GBOOST were assigned to the corresponding genes. Subsequently the gene pairs that contained at least one target gene were screened for further analysis. We also used permutation tests to reduce the risk of false-positives (calculating 10,000 times in total). The results of the chi-square test can be further corrected using *permutation* tests (FDR < 0.005). The overall workflow of our method is presented as Figure 3.

## 4. Conclusions 

The emergence and spread of resistant tuberculosis has become the most significant threat to global TB control. Drug resistance patterns may vary widely from single-drug to multiple-drug resistance, and the emergence of transmissible drug resistance is associated with multiple genetic mutations. To reveal the mechanisms of drug resistance and to develop better control strategies, we identified gene pairs associated with *Mtb* drug resistance.

All of the associations of SNP pairs were screened and assigned to the corresponding genes. Subsequently, to reveal the potential resistance mechanisms of the *Mtb* strains, the obtained gene pairs were filtered by drug targets and non-synonymous mutations. The obtained non-synonymous mutations in the drug target genes were compared with the reported experiments, and the results validated the effectiveness of our method. Many drug resistance-associated gene pairs that contained a target gene were identified: one for INH, one for RMP, four for EMB and five for ETH. Further gene pair analyses revealed that the detected *katG-PPE54* and *rpoB-PPE54* gene pairs are associated with INH and RMP resistance, respectively. The *embA-PPE68* and *embB-PPE54* gene pairs play possible roles in *Mtb* resistance to EMB. The joint mutations of *katG-PPE54* gene pairs would bring *Mtb* resistance to ETH.

In summary, this study indicated the potential of bioinformatics methods in predicting drug resistance-associated gene pairs and clarifying the resistance mechanisms of anti-tuberculosis drugs. Moreover, the present approach is also useful for exploring the mechanisms of drug resistance for other microorganisms.

## Figures and Tables

**Table 1:** Anti-tuberculosis drug classification, with inclusion of new compounds under evaluation ^a^.

Group	Drug
Group 1: First-line oral agents	isoniazid; rifampicin; ethambutol; pyrazinamide
Group 2: Injectable agents	kanamycin; amikacin; capreomycin; streptomycin
Group 3: Fluoroquinolones	ofloxacin; levofloxacin; moxifloxacin; gatifloxacin
Group 4: Oral bacteriostatic second-line agents	*p*-aminosalicylic acid; cycloserine; terizidone; ethionamide; protionamide
Group 5: Agents with unclear efficacy	clofazimine; linezolid; amoxicillin; imipenem; clarithromycin; thioacetazone

^a^ According to Reference [6].

**Table 2:** Anti-tuberculosis drug resistance-associated genes ^a^.

Drug	Genes Involved in Resistance
Capreomycin (CPM)	*rrs*, *tlyA*
Ethambutol (EMB)	*embA*, *embB*, *embC*
Ethionamide (ETH)	*inhA*, *katG*
Isoniazid (INH)	*katG*, *inhA*, *ahpC*, *fabG*, *fadE24*
Kanamycin (KAN)	*rpsL rrs*
Ofloxacin (OFX)	*gyrA*, *gyrB*
Rifampicin (RMP)	*rpoA*, *rpoB*, *rpoC*
Streptomycin (STR)	*gidB*, *rpsL rrs*

^a^ According to reference [13].

**Table 3:** The obtained gene pairs containing target genes in dataset 1.

Gene ID (Target Name)	Gene ID (Gene Name)	SNP1	SNP2	*Chi-Square* Test	*Permutation* Test
Rv1908c (*katG*) ^a^	Rv3343c (*PPE54*) ^a^	rs_5245	rs_8894	1.69 × 10^−3^	<0.0001
Rv0667 (*rpoB*) ^b^	Rv3343c (*PPE54*) ^b^	rs_2019	rs_8894	1.28 × 10^−4^	<0.0001

^a^ associated with isoniazid resistance; ^b^ associated with rifampicin resistance.

**Table 4:** The obtained gene pairs containing target genes in dataset 2.

Gene ID (Target Name)	Gene ID (Gene Name)	SNP1	SNP2	*Chi-Square* Test	*Permutation* Test
Rv3794 (*embA*) ^a^	Rv3873 (*PPE68*) ^a^	rss_17400	rss_17926	1.98 × 10^−3^	0.0016
Rv3795 (*embB*) ^a^	Rv3343c (*PPE54*) ^a^	rss_17410	rss_15182	2.29 × 10^−3^	<0.0001
Rv3795 (*embB*) ^a^	Rv3343c (*PPE54*) ^a^	rss_17411	rss_15181	8.55 × 10^−4^	0.0004
Rv3795 (*embB*) ^a^	Rv3343c (*PPE54*) ^a^	rss_17411	rss_15182	2.19 × 10^−3^	<0.0001
Rv1908c (*katG*) ^b^	Rv3343c (*PPE54*) ^b^	rss_8886	rss_15184	2.16 × 10^−3^	0.0023
Rv1908c (*katG*) ^b^	Rv0576 (*transcriptional regulator*) ^b^	rss_8886	rss_3040	1.86 × 10^−3^	<0.0001
Rv1908c (*katG*) ^b^	Rv0529 (*ccsA*) ^b^	rss_8886	rss_2852	1.78 × 10^−3^	0.0038
Rv1908c (*katG*) ^b^	Rv3554 (*fdxB*) ^b^	rss_8886	rss_16297	1.20 × 10^−3^	0.0009
Rv1908c (*katG*) ^b^	Rv0575c (*oxidoreductase*) ^b^	rss_8900	rss_3037	1.80 × 10^−3^	0.0009

^a^ associated with ethambutol resistance; ^b^ associated with ethionamide resistance.

**Table 5:** The non-synonymous mutations in the target genes from the two datasets.

Drug	SNP	SNP Loci	Gene ID	Target	Base Mutation	Amino Acid Mutation	Previously Reported
INH ^a^	rs_5245	2154724	Rv1908c	KatG	CGG > CTG	R463L	[19]
EMB ^b^	rss_17410	4247709	Rv3795	*embB*	AAC > ACC	N399T	–
EMB ^b^	rss_17411	4247729	Rv3795	*embB*	GGC > AGC	G406S	[16,21]
ETH ^c^	rss_8886	2154724	Rv1908c	KatG	CGG > CTG	R463L	–
ETH ^c^	rss_8900	2155648	Rv1908c	KatG	TAC > TGC	Y155C	[16]

^a^ isoniazid; ^b^ ethambutol; ^c^ ethionamide.

## Supplementary Materials

Supplementary materials can be found at www.mdpi.com/1422-0067/17/9/1417/s1.
