# Supplementary material for: Bioinformatics Identification of Drug Resistance-Associated Gene Pairs in Mycobacterium tuberculosis
Source: Int J Mol Sci. 2016 Aug 27;17(9):1417. doi: 10.3390/ijms17091417 (PMC5037696; doi:10.3390/ijms17091417)
Supplement: Supplementary file 1 [file ijms-17-01417-s001.pdf]

Supplementary Materials: Bioinformatics Identification of Drug Resistance-Associated Gene Pairs in *Mycobacterium tuberculosis*

Ze-Jia Cui, Qing-Yong Yang, Hong-Yu Zhang, Qiang Zhu and Qing-Ye Zhang

Table S1. The summarized results by GBOOST (The Hong Kong University, Clear Water Bay, Kowloon, Hong Kong, China) based on the dataset 1.

Sample	EMB [a]	INH [b]	RMP [c]	STR [d]
SNP pairs	1407	3057	3925	2018
Gene pairs	1215	2597	3328	1636

[a] ethambutol; [b] isoniazid; [c] rifampicin; [d] streptomycin.

Table S2. The summarized results by GBOOST based on the dataset 2.

Sample	CPM [a]	EMB [b]	ETH [c]	INH [d]	KAN [e]	OFX [f]	RMP [g]	STR [h]
SNP pairs	786	840	2499	269	871	722	269	1244
Gene pairs	695	694	2002	219	761	620	219	969

[a] capreomycin; [b] ethambutol; [c] ethionamide; [d] isoniazid; [e] kanamycin; [f] ofloxacin; [g] rifampicin; [h] streptomycin.

Table S3. Anti-tuberculosis drug targets information from DrugBank [a].

Drug	Targets (Abbreviation)
Capreomycin (CPM)	70S ribosome
Ethambutol (EMB)	probable arabinosyltransferase C (embC)
	probable arabinosyltransferase B (embB)
	probable arabinosyltransferase A (embA)
Ethionamide (ETH)	catalase-peroxidase (KatG)
	enoyl-(acyl-carrier-protein) reductase (NADH) (InhA)
Isoniazid (INH)	catalase-peroxidase (KatG)
	enoyl-(acyl-carrier-protein) reductase (NADH) (InhA)
	cytochrome P450 2C8
	cytochrome P450 1A2
	cytochrome P450 3A4
	cytochrome P450 2C19
	dihydrofolate reductase
Kanamycin (KAN)	30S ribosomal protein S12
	16S rRNA
Ofloxacin (OFX)	DNA gyrase subunit A
	DNA topoisomerase 4 subunit A
	DNA topoisomerase 2-α

Table S3. *Cont.*

Drug	Targets (Abbreviation)
Rifampicin (RMP)	DNA-directed RNA polymerase subunit β
	DNA-directed RNA polymerase subunit β
	nuclear receptor subfamily 1 group I member 2
	solute carrier organic anion transporter family member 1B1
	solute carrier organic anion transporter family member 1A2
	solute carrier organic anion transporter family member 1B3
	Lanosterol 14-α demethylase
	Serum albumin
	solute carrier organic anion transporter family member 2B1
Streptomycin (STR)	30S ribosomal protein S12
	16S rRNA
	protein-arginine deiminase type-4

[a] http://www.drugbank.ca.
